# Cell death in *Leishmania*

**DOI:** 10.1051/parasite/2019071

**Published:** 2019-12-11

**Authors:** Louise Basmaciyan, Magali Casanova

**Affiliations:** 1 UMR PAM A, Valmis Team 2 rue Angélique Ducoudray BP 37013 21070 Dijon Cedex France; 2 Aix-Marseille University, CNRS, LISM, Institut de Microbiologie de la Méditerranée 13402 Marseille Cedex 09 France

**Keywords:** *Leishmania*, Cell death, Phenotype, Role, Pathways

## Abstract

Leishmaniases still represent a global scourge and new therapeutic tools are necessary to replace the current expensive, difficult to administer treatments that induce numerous adverse effects and for which resistance is increasingly worrying. In this context, the particularly original organization of the *Leishmania* parasite in comparison to higher eukaryotes is a great advantage. It allows for the development of new, very specific, and thus non-cytotoxic treatments. Among these originalities, *Leishmania* cell death can be cited. Despite a classic pattern of apoptosis, key mammalian apoptotic proteins are not present in *Leishmania*, such as caspases, cell death receptors, and anti-apoptotic molecules. Recent studies have helped to develop a better understanding of parasite cell death, identifying new proteins or even new apoptotic pathways. This review provides an overview of the current knowledge on *Leishmania* cell death, describing its physiological roles and its phenotype, and discusses the involvement of various proteins: endonuclease G, metacaspase, aquaporin Li-BH3AQP, calpains, cysteine proteinase C, LmjHYD36 and Lmj.22.0600. From these data, potential apoptotic pathways are suggested. This review also offers tools to identify new *Leishmania* cell death effectors. Lastly, different approaches to use this knowledge for the development of new therapeutic tools are suggested: either inhibition of *Leishmania* cell death or activation of cell death for instance by treating cells with proteins or peptides involved in parasite death fused to a cell permeant peptide or encapsulated into a lipidic vector to target intra-macrophagic *Leishmania* cells.

## Abstract (fr)

Alors que les leishmanioses représentent toujours un fléau mondial, de nouveaux outils thérapeutiques sont nécessaires pour remplacer les traitements actuels qui sont chers, difficiles à administrer, qui induisent de nombreux effets secondaires et pour lesquels la résistance est de plus en plus inquiétante. Pour cela, l’organisation très originale du parasite *Leishmania* par rapport aux eucaryotes supérieurs est un grand avantage. En effet, cela permet le développement de nouveaux traitements très spécifiques et donc non cytotoxiques. Parmi ces originalités, la mort cellulaire de *Leishmania* peut être citée. Malgré un aspect classique d’apoptose, les protéines apoptotiques clefs des mammifères ne sont pas présentes chez *Leishmania*, telles que les caspases, les récepteurs de mort et les molécules anti-apoptotiques. Des études récentes ont participé à une meilleure compréhension de la mort cellulaire du parasite, identifiant de nouvelles protéines ou même de nouvelles voies apoptotiques. Cette revue fait le point sur l’état actuel des connaissances sur la mort cellulaire de *Leishmania*, décrivant ses rôles physiologiques et son phénotype et discutant l’implication de différentes protéines : endonucléase G, métacaspase, aquaporine Li-BH3AQP, calpaïnes, cystéine protéinase C, LmjHYD36 et Lmj.22.0600. À partir de ces données, différentes voies apoptotiques potentielles sont suggérées. Cette revue fournit également des outils pour identifier de nouveaux effecteurs de la mort cellulaire de *Leishmania*. Pour finir, différentes approches pour utiliser ces connaissances pour le développement de nouveaux outils thérapeutiques sont suggérées : soit inhibition de la mort cellulaire de *Leishmania*, soit activation de celle-ci par exemple en traitant les cellules avec des protéines/peptides impliqués dans la mort du parasite fusionnés à un peptide pénétrant dans les cellules ou encapsulés dans un vecteur lipidique pour cibler les cellules de *Leishmania* intra-macrophagiques.

**Abbreviations**
ACD: Accidental Cell Death
CALP: CAlpain-Like Proteins
CPC: Cysteine Proteinase C
MCA: Metacaspase
PCD: Programmed Cell Death
PI: Propidium Iodide
RCD: Regulated Cell Death
SKCRP: Small Kinetoplastid Calpain-Related Proteins
WHO: World Health Organization

## Introduction: *Leishmania* parasites

Parasites of the *Leishmania* genus, which are responsible for leishmaniases, are flagellated protozoa of the Trypanosomatidae family like *Trypanosoma brucei*, the causal agent of human African trypanosomiasis, and *T. cruzi*, the agent of Chagas disease. Leishmaniases are considered by the World Health Organization (WHO) to be a neglected tropical disease, mainly affecting low-income people worldwide. Between 700,000 and 1 million infections are estimated worldwide, causing between 20,000 and 30,000 deaths per year (Global Health Observatory data from WHO, 2018). In 2017, 94% of the new cases reported to the WHO occurred in seven countries: Brazil, Ethiopia, India, Kenya, Somalia, South Sudan and Sudan (WHO reports). Three main types of leishmaniasis are described: cutaneous, mucocutaneous and visceral leishmaniasis, also known as kala-azar. Leishmaniases are a major health problem because of the absence of satisfactory treatments, the adverse effects of treatments, their cost, their mode of administration which is mostly parenteral, limiting their use in developing countries, and the emergence of drug-resistant strains.

*Leishmania* parasites are transmitted to mammals by the bite of a female sand fly. In the digestive tract of the vector, parasites proliferate as mobile flagellated parasites called promastigotes: procyclic promastigotes within the midgut and virulent metacyclic promastigotes in the insect proboscis [9, 20]. After inoculation to the mammalian host, promastigotes are taken up by phagocytic cells where they transform into an immobile form with a reduced flagellum called the amastigote form.

## Cell death in general

Contrary to what one might think, the limit between life and death is not so clear. To overcome this, the Nomenclature Committee on Cell Death proposed, in 2015, to consider a cell to be dead if (i) the cell has lost its plasma membrane integrity, or if (ii) the cell, including the nucleus, has undergone complete fragmentation into what is usually termed “apoptotic bodies”, or if (iii) cell fragments have been phagocytized by a nearby cell *in vivo* [40]. Currently, two main types of cell death are described: Accidental Cell Death (ACD) and Regulated Cell Death (RCD) [28]. ACD is induced by harsh physical, chemical or mechanical damage such as, for example, high temperatures, high pressure, potent detergents, extreme pH variations or shearing [28]. This type of cell death is virtually immediate and does not involve specific molecular machinery [28]. As a consequence, no genetic construction and no specific drug can be used to activate or inhibit ACD. On the contrary, RCD involves molecular machinery that is genetically encoded, allowing genetic and pharmacologic manipulations to alter this type of cell death [28]. RCD appears in two circumstances: (i) when the cell is facing micro-environmental disruptions, and (ii) during embryonic development, tissue homeostasis and immune responses; this physiological type of RCD being termed Programmed Cell Death (PCD) [28].

The terms necrosis, apoptosis and autophagy are classically described based on cell morphology. Necrosis is morphologically characterized by a gain in cell volume, organelle swelling, loss of plasma membrane integrity and, as a consequence, loss of intracellular contents [41]. For a long time, necrosis was considered a form of accidental non-controlled cell death, inducing ACD. However, it is now described as a potential RCD inducer since it can be finely regulated owing to specific signal transduction pathways [41].

The term “apoptosis”, coined by Kerr et al. [39], is, by definition, a type of cell death that is characterized by several morphological features among which cell rounding, retraction of pseudopods, cell shrinkage, chromatin condensation, nuclear fragmentation, few ultrastructural modifications of cytoplasmic organelles, plasma membrane modifications with maintenance of its integrity, membrane blebbing that culminates in the formation of apoptotic bodies and, *in vivo*, phagocytosis by nearby cells [40]. Apoptosis induces RCD and it is often wrongly confused with PCD which is, as previously said, a physiological form of RCD that occurs in the context of embryo development and tissue homeostasis [30]. Classically, apoptosis is described as being initiated by intracellular or extracellular stimuli, involving the intrinsic or extrinsic apoptosis pathway respectively, two signal transduction pathways that are interconnected. The intrinsic pathway involves pro- and anti-apoptotic molecules of the Bcl-2 family and relies on mitochondrial outer membrane permeabilization that results in the release of molecules from mitochondria, such as cytochrome c, and, *in fine*, to the activation of specific enzymes called caspases [26]. The extrinsic pathway involves cell death receptors inserted in the plasma membrane as well as caspases and molecules of the Bcl-2 family [12]. It should be noted that this view of the apoptotic signal transduction pathways is simplistic. In fact, the term “apoptosis” hides high biochemical, functional and immunological diversity. Several apoptotic subtypes, while morphologically similar, can be induced by different biochemical pathways and may result in different consequences, for instance concerning recognition by the immune system [41]. In the same way, while ACD consistently presents a necrotic morphology (cell disintegration by loss of plasma membrane integrity), RCD can present a wide range of morphologies and can result from different signal transduction pathways, including regulated necrosis and apoptosis [15]. To define these different types of RCD, based on their biochemical and molecular characterization, many terms have been introduced, such as, for instance, extrinsic or intrinsic apoptosis, mitochondrial permeability transition-driven necrosis, necroptosis, ferroptosis or pyroptosis [29].

Concerning macro-autophagy, here confused with autophagy, it is a cell survival process that allows the cell to survive nutrient or growth factor deprivation [45]. More precisely, it is an intracellular catabolic process that sequesters cytosol and organelles in double-membrane vesicles called autophagosomes for fusion with and degradation into lysosomes [45]. Amino acids generated are recycled and used for protein synthesis. Some authors have introduced the term “autophagic cell death”. However, as clearly defined by the Nomenclature Committee on Cell Death, it does not correspond to a type of cell death that is induced by autophagy but a type of cell death with autophagy, that is to say where autophagosomes appear [30]. As a consequence, autophagic cell death is a form of cell death that can be inhibited or delayed by pharmacologic or genetic inhibition of the autophagic molecular machinery [40].

## Physiological roles of *Leishmania* RCD

**Figure 1:**
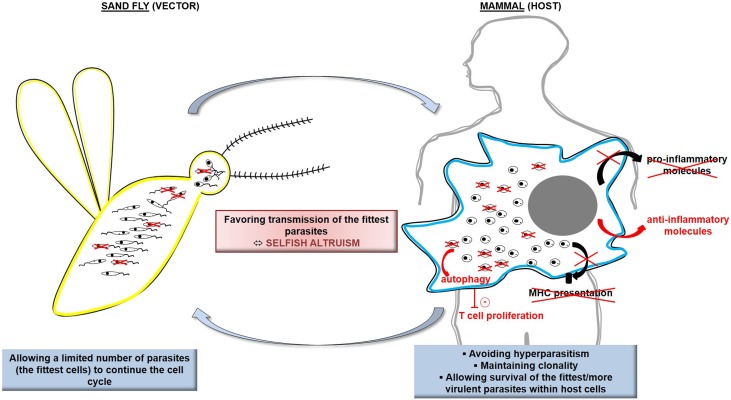
Schematic representation of the physiological roles of *Leishmania* RCD. In the sand fly vector, *Leishmania* RCD allows a limited number of parasites, among the fittest, to continue the cell cycle, which is of particular interest because of the limited resources within the vector digestive tract. In the mammalian host, *Leishmania* RCD induces elimination of damaged/unfit cells, inhibition of the secretion of pro-inflammatory molecules and, on the contrary, secretion of anti-inflammatory molecules by the infected macrophage, inhibition of parasite antigen presentation, and activation of the macrophage autophagy machinery which negatively regulates T cell proliferation. Ultimately, this makes it possible to avoid hyperparasitism, to maintain *Leishmania* clonality, and to ensure survival of the fittest more virulent parasites. As a conclusion, *Leishmania* RCD appears as a selfish altruism, favoring transmission of the fittest parasites.

The controlled suicide of cells in multicellular organisms is a clearly accepted process, allowing the disposal of superfluous cells during organism development or damaged cells that would compromise the whole organism [26]. In unicellular organisms such as protozoan parasites, the existence of cell suicide has long been a matter of debate until the demonstration of several roles of RCD, *in vivo*, in the biology of the organism. In *Leishmania*, RCD appears to regulate the parasitic population in response to the limited resources in the sand fly gut [43, 72]. For instance, promastigotes that are not differentiated into the metacyclic infectious form would enter RCD, which would be beneficial for metacyclics since procyclics would not consume essential nutrients found in restricted quantities in the sand fly gut [21]. As a consequence, RCD appears to be an altruistic mechanism for selection of parasites suitable for disease transmission. RCD also appears to regulate parasite density within the host, to avoid hyperparasitism that would kill the host and thus prevent parasite transmission [14, 47]. RCD seems to maintain clonality of the parasitic population, removing non-suitable cells and thus ensuring propagation of the fittest, more virulent cells [63]. Furthermore, it has been demonstrated that phagocytosis of apoptotic *Leishmania* cells induces secretion, by macrophages, of anti-inflammatory molecules such as IL-10 and TGF-β or lipids such as lipoxin A4; on the contrary, it induces inhibition of the secretion of pro-inflammatory cytokines such as TNF-α and lipids such as leukotriene B4 [67, 70]. In case of disposal of apoptotic parasites from a virulent population, *Leishmania* cells do not survive within phagocytic cells *in vitro* and lose their ability to induce the disease *in vivo* [67, 70]. Phagocytosis of apoptotic cells also appears to decrease presentation of parasite antigens by macrophages [74]. Lastly, apoptotic cells activate the autophagic machinery of the host cells, reducing T cells proliferation and thus favoring parasite survival [19]. In other words, apoptotic *Leishmania* parasites allow, in an altruistic manner, the intracellular survival of the fittest parasites. The term “selfish altruism” has been introduced for qualifying RCD in protozoan parasites: an altruistic phenomenon implemented for the whole population that is clonal [24]. Figure 1 illustrates these physiological roles of *Leishmania* RCD.

## Apoptosis phenotype in *Leishmania*

In *Leishmania*, different stimuli induce a phenotype similar to mammalian apoptosis *in vitro*, such as heat shock, reactive oxygen species (nitric oxide, hydrogen peroxide), anti-infectious molecules (such as novobiocin or amphotericin B), anti-cancer molecules (such as miltefosine or doxorubicin), molecules from plants (such as whitaferin A, camptothecin or aloe vera extracts) or lipids (such as edelfosine) (summary in [38, 64]). These stimuli induce cell rounding, cell shrinkage, plasma membrane changes with maintenance of its integrity, mitochondrial modifications, chromatin condensation, nuclear fragmentation and DNA fragmentation [38]. Extensive inhibition of mRNA translation is also observed during *Leishmania* cell death, this inhibition being correlated to the fragmentation of antisense RNA complementary to ribosomal RNA [54]. As apoptosis is defined on cell morphology, the term “apoptosis” should be used for this type of *Leishmania* cell death and other words like “apoptosis-like cell death” or “programmed cell death” should be avoided.

To be more precise, molecular characterization of *Leishmania* cell death should be carried out. However, the limited knowledge of proteins involved in *Leishmania* cell death prevents this approach. As a consequence, *Leishmania* apoptosis is still described on cell morphology and we think that new terms, such as microptosis [23], should be avoided while transduction signals are not known. Furthermore, demonstrating that a stimulus induces *Leishmania* apoptosis needs to take into account *Leishmania* particularities. For instance, no membrane blebbing and no apoptotic bodies have been observed in *Leishmania* cells undergoing apoptosis [38]. Furthermore, the small size of the parasite, and consequently of its nucleus, makes it difficult to show chromatin condensation by fluorescence microscopy. In addition, Annexin V is not an apoptosis marker in *Leishmania* [71]. In higher eukaryotes, this molecule binds to phosphatidylserine, a phospholipid naturally present in the inner plasma membrane leaflet that switches to the outer leaflet during apoptosis [50]. In *Leishmania* parasites, while no phosphatidylserine could be detected, Annexin V can bind to other phospholipid classes [71]. As a consequence, Annexin V staining is a marker of plasma membrane modifications but not an apoptosis marker in *Leishmania*. On the contrary, a new apoptotic marker has been described in *Leishmania*: calcein. Interestingly, used in combination with propidium iodide (PI), calcein allows discrimination between healthy, early apoptotic, late apoptotic and necrotic cells [4]. Lastly, since *Leishmania* parasites lack caspases, the key enzymes of metazoan apoptosis [59], no conclusions can be drawn from experiments highlighting caspase-like activity until the molecular characterization of the enzymes involved.

Moreover, to demonstrate *Leishmania* apoptosis, specific markers should be used. Clearly, morphologic changes, such as cell rounding or cell shrinkage, cannot be used as a specific apoptotic marker since it can appear under other stress conditions. In the same manner, mitochondrial depolarization, preceded by transient hyperpolarization in *Leishmania* [38], and the loss of plasma membrane integrity are not specific apoptosis markers.

Apoptosis must also be distinguished from other processes, specifically autophagy. Despite their opposite functions, cell death and the survival process autophagy are closely linked: besides their reciprocal inhibition, each process can activate the other [48]. Autophagy can in particular induce apoptosis by degrading parts of the cell after sequestration in autophagosomes and degradation within lysosomes, or by activating the apoptotic pathway [48]. In *Leishmania*, autophagic cells enter apoptosis in the absence of nutrients [7]. This close relationship often establishes confusion between the apoptotic and autophagic processes, some authors talking about apoptosis in response to nutrient deprivation or during the stationary phase of *Leishmania*, while these stimuli induce autophagy [10].

**Figure 2:**
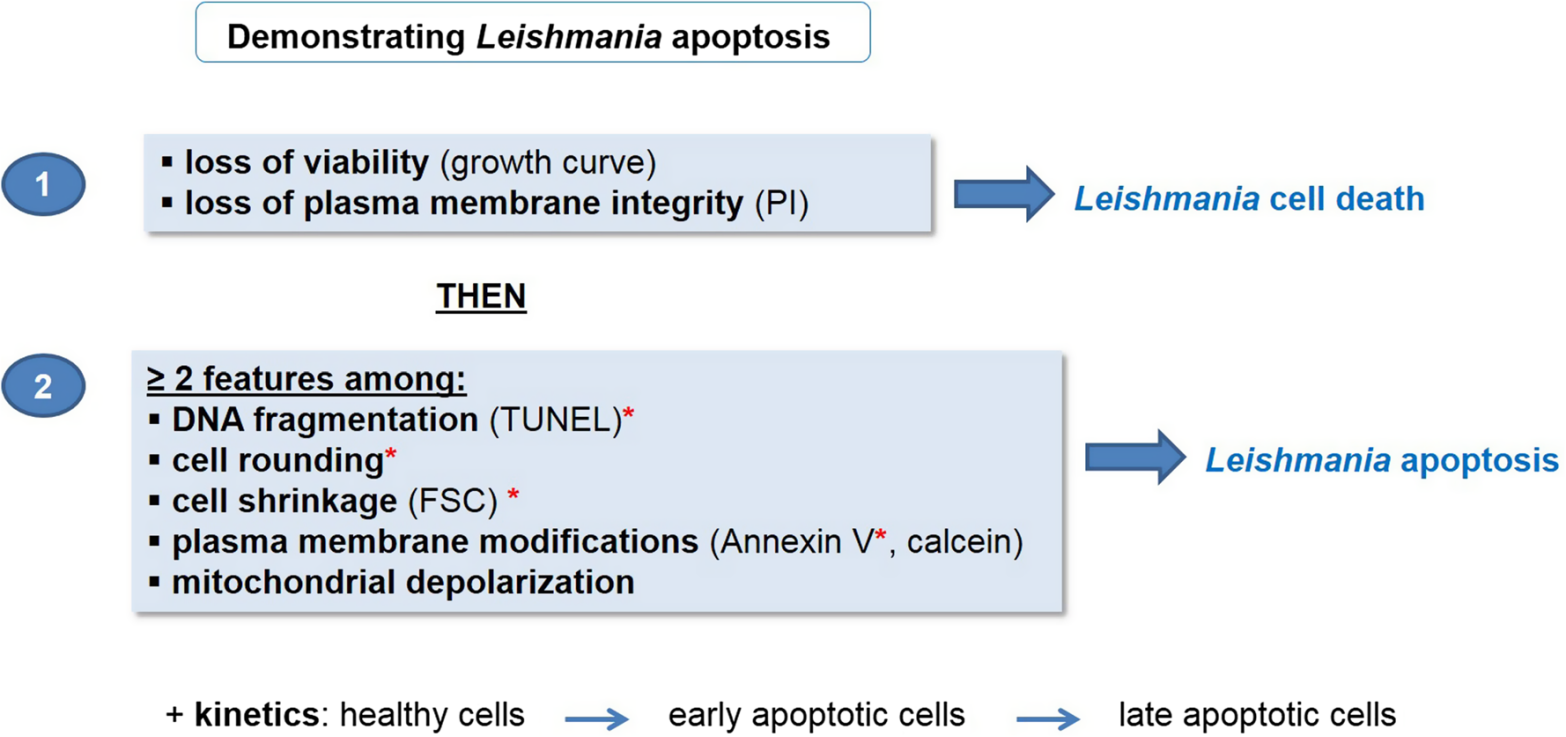
Proposed procedure to demonstrate *Leishmania* apoptosis. To demonstrate *Leishmania* apoptosis, we propose to first show cell death in general by assessing the loss of viability by establishing a growth curve and the loss of plasma membrane integrity by PI staining. Second, the type of cell death should be evaluated. In case of apoptosis, at least two apoptotic markers should be highlighted, among which DNA fragmentation (by a TUNEL assay which is easier than showing a DNA ladder), cell rounding, cell shrinkage (by evaluating Forward Side Scatter (FSC) by flow cytometry), plasma membrane modifications (by Annexin V or calcein staining) and mitochondrial depolarization. Asterisks indicate markers that are not present in autophagic cells. Apoptosis kinetics should also be evaluated, showing entry of healthy cells into early apoptosis and then into late apoptosis, to distinguish apoptosis from necrosis.

As a consequence, we propose unified criteria for the definition of *Leishmania* apoptosis as has recently been done in yeast [15] (Fig. 2). We propose to first demonstrate *Leishmania* cell death by evaluating loss of viability by a growth curve and loss of plasma membrane integrity by PI staining. As a second step, we recommend defining the type of cell death, here apoptosis, by showing the presence of at least two apoptotic markers among DNA fragmentation, cell rounding, cell shrinkage, plasma membrane modifications and mitochondrial depolarization. To evaluate DNA fragmentation, a TUNEL assay should be preferred to an experiment showing the DNA ladder because the latter could be difficult to do [38]. DNA fragmentation, cell rounding, cell shrinkage and plasma membrane modifications evaluated by Annexin V staining make it possible to distinguish apoptosis from autophagy, since these features are not present in autophagic cells [7]. Furthermore, apoptosis kinetics should be carried out to evaluate the entry of healthy cells into early apoptosis and then into late apoptosis. Late apoptotic cells being indistinguishable from necrotic cells, this ensures that the cells die from apoptosis. To supplement this approach, contrary to what is recommended in yeast [15] and metazoans [29], the regulatory network cannot be assessed in *Leishmania* since the executioner proteins and the metabolic pathways involved are currently unknown.

## Proteins involved in *Leishmania* apoptosis

In spite of the clear existence of *Leishmania* apoptosis and several studies on this phenomenon, it remains largely unknown. This is due to the fact that key proteins involved in mammalian apoptosis could not be identified in *Leishmania*, even bio-informatically. More precisely, caspases, cell death receptors and anti-apoptotic molecules could not be identified [64]. Recently, several proteins that seem to be involved in *Leishmania* cell death have been identified. Below, we describe the proteins that are currently known.

### The endonuclease G (EndoG)

EndoG has been well characterized in *C. elegans* and mammals: it is a cell death effector that localizes in the mitochondria and translocates to the nucleus in response to apoptotic stimuli where it degrades DNA [46, 56, 66]. Concerning *Leishmania*, EndoG has been identified and well-characterized in two species, showing a similar function as in other organisms: LdBPK_100600.1 in *L. donovani* and LINF_100012900 in *L. infantum* [11, 31, 61]. More precisely, in both species, the enzyme possesses nuclease activity *in vitro*, depending on the three conserved catalytic amino acid residues (R, G and H) [11, 31, 61]. Under normal conditions, the enzyme is localized in the mitochondrion of promastigotes as well as of axenic amastigotes [11, 31, 61], where it appears to be essential for parasite survival [62]. In the mitochondrion, where the pH is relatively high, a small amino acid sequence inhibits the nuclease activity [53]. Different apoptotic stimuli, such as H_2_O_2_ or edelfosine, induce EndoG translocation from the mitochondrion to the nucleus where it forms a complex, on the one hand, with the Flap endonuclease-1 FEN-1 and, on the other, with the nuclease TatD [11]. This results in DNA degradation through endonuclease and exonuclease activities, notably on single-stranded DNA [11, 31, 61, 62]. As a consequence, despite unique properties of *Leishmania* EndoG compared to other EndoG [53], this enzyme is a well-characterized cell death effector in the parasite.

### Metacaspase

**Figure 3:**
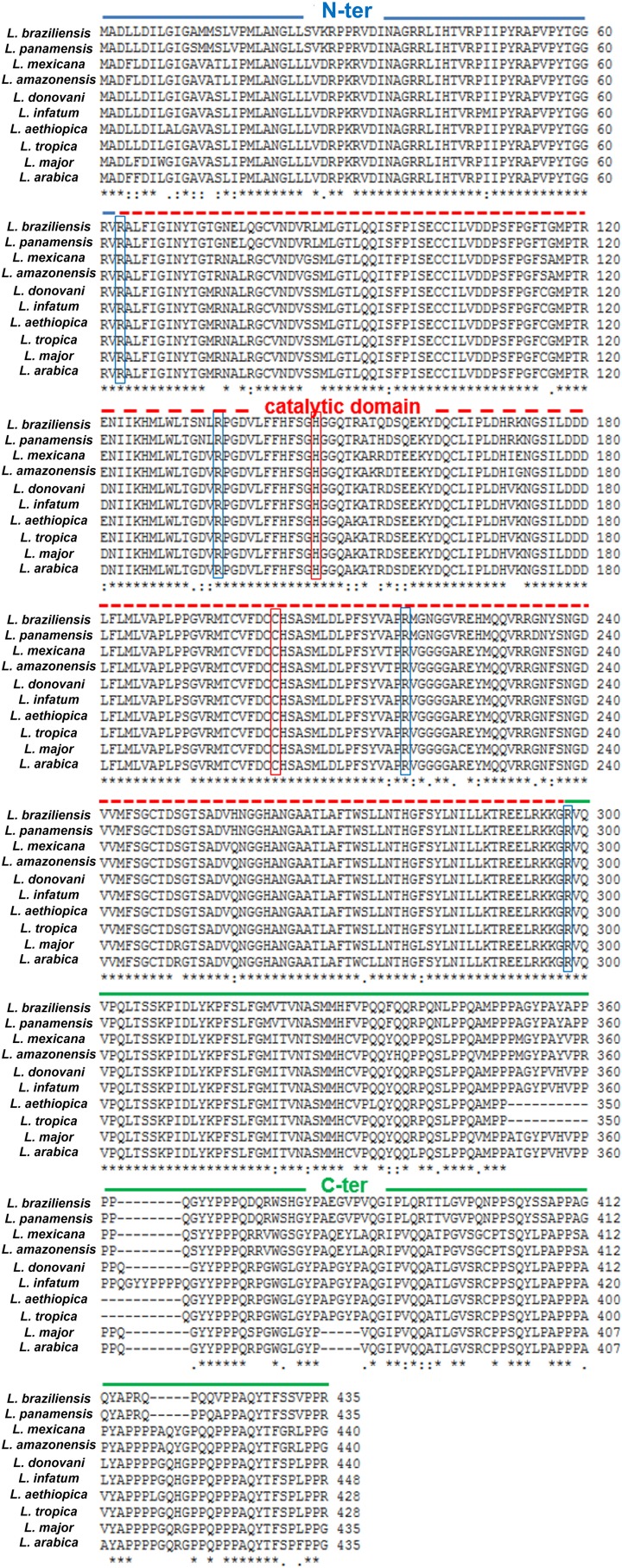
Alignment of the amino-acid sequences of the MCA orthologs in different *Leishmania* species. The N-terminal domain, which contains a mitochondrial localization signal, from amino-acids 1 to 62, is indicated in blue; the catalytic domain, containing the catalytic dyad histidine 147/cysteine 202 (boxed in red), is indicated by a dashed red line; the proline-rich C-terminal domain is indicated by a green line. Demonstrated cleavage sites are boxed in blue: R63, R136, R218 and R298.

Metacaspases are cysteine peptidases that have been identified in fungi, plants and protozoa. They possess a caspase-like domain homologous to the p20 subunit of caspases, with the catalytic dyad histidine/cysteine, and a C-terminal proline-rich domain homologous to the p10 subunit of caspases [35, 65, 68, 73]. However, while caspases are specific to substrates containing an aspartate at P1 position, metacaspases are specific to substrates with an arginine or lysine [35, 52, 68]. Metacaspases have been studied in different *Leishmania* species where they share a high percentage of identity, the catalytic dyad histidine 147 and cysteine 202 and cleavage sites (notably arginine at position 63, 136, 218 and 298) (Fig. 3).

In *L. major*, the metacaspase, termed LmjMCA, encoded by the *LmjF.35.1580* gene, is expressed in the different parasite forms: in the logarithmic and stationary promastigotes, as well as in the amastigotes [2, 35]. It is localized mainly in the cytoplasm, but also in the single mitochondrion thanks to an N-terminal localization signal [2, 73]. More precisely, localization of LmjMCA is modified during the cell cycle, being distributed in the whole cytoplasm during interphase, and being associated with the kinetoplast, the mitochondrial DNA, during organelle segregation or with the nuclear mitotic spindle during mitosis [2]. One hypothesis is that mitochondrial translocation is prevented by a short sequence of LmjMCA, explaining its cytoplasmic localization [73]. LmjMCA has no caspase-like activity but it possesses trypsine-like activity towards arginine at position P1 that depends on the catalytic dyad H147/C202 [35, 49]. However, it is involved in apoptosis as caspases. Indeed, the expression of LmjMCA in *S. cerevisiae* yeasts lacking the endogenous metacaspase YCA1 restores the apoptotic phenotype after oxidative stress, this restoration being dependent on the presence of the LmjMCA catalytic dyad [35]. Furthermore, overexpression of LmjMCA induces *L. major* apoptosis after heat shock, oxidative stress or the addition of an anti-leishmanial drug (miltefosine or curcumin) [73], while its deletion inhibits miltefosine- and curcumin-induced apoptosis [16]. More precisely, apoptotic stimuli induce auto-processing of LmjMCA depending on the catalytic dyad, releasing the catalytic domain, at least from amino-acids 136 to 218, containing the catalytic dyad His147/Cys202, after cleavage of the C-terminal domain [35, 73]. It seems that the catalytic domain released induces apoptosis *via* the catalytic cleavage of substrates, while the proline-rich C-terminal domain that has been released during apoptosis interacts with proteins involved in stress regulation and apoptosis such as the mitogen-activated protein kinase LmaMPK7 and the calpain-like protease LmCALP27.2 [16]. It has also been shown that LmjMCA is involved in the cell survival process autophagy in *L. major* [16], underlying a broader role of LmjMCA than only in cell death as already demonstrated for caspases [22, 37, 42].

In *L. donovani*, two metacaspases have been described: LdMC1 (DQ367530) and LdMC2 (DQ367531) [44]. However, LdMC2 does not appear in the TriTryp database (https://tritrypdb.org/tritrypdb/), it has the same sequence as LmjF.35.1580 and experiments have been done with LdMC1 or LdMC antibodies that do not discriminate between both proteins, raising the question of the nature of LdMC2. Concerning LdMC1, it is expressed in promastigotes and axenic amastigotes [44]. Contrary to LmjMCA, it seems that it is not processed in wild-type and apoptotic conditions [44]. It is localized in acidocalcisomes, where acidic pH may inhibit its activity [44]. It has no caspase-like activity, explaining the absence of effects of caspase inhibitors, but possesses trypsine-like activity, cleaving substrates containing an arginine or a lysine at position P1 [44]. Lastly, LdMC1 seems to be involved in *L. donovani* H_2_O_2_-induced apoptosis after release from the acidocalcisomes [44]. Some authors indicate that LdMC1 is involved in *L. donovani* survival [60]. However, the use of markers that are not specific to apoptosis and/or experiments that are difficult to interpret underlines the need for further experiments to draw such conclusions.

The third and last *Leishmania* species in which the role of metacaspase in cell death has been studied is *L. mexicana*, which contains a single metacaspase here termed MCA encoded by the *LmxM.34.1580* gene. This metacaspase, mainly localized in the cytoplasm, seems involved in *L. mexicana* miltefosine-induced apoptosis at least in promastigotes, cells lacking the corresponding gene being less susceptible to the pro-apoptotic drug miltefosine [17]. However, MCA-deficient *L. mexicana* cells have no different susceptibility towards oxidative stress or towards the camptothecin drug [17]. In *L. mexicana*, MCA is also a negative regulator of intracellular amastigote proliferation [17].

These data on metacaspases in different *Leishmania* species, summarized in Table 1, are not necessarily mutually exclusive and do not preclude similar roles in the different species. Metacaspase could be involved in the *Leishmania* cell cycle and regulation of the intracellular amastigote proliferation irrespective of the species, and could be recruited in the presence of an apoptotic stimulus to take part in cell death.

**Table 1:** Characterization of the metacaspase protein in different *Leishmania* species. ND: not determined.

Species	Gene ID	Wild-type expression	Processing	Localization	Enzymatic activity	Apoptotic stimuli inducing MCA activity	Role other than in apoptosis	References
*L. major*	*LmjF.35.1580*	In promastigotes and amastigotes	Auto-processing	Depends on the cell cycle phase; cytoplasm and mitochondrion; associated with the kinetoplast and the mitotic spindle	Trypsine-like activity (no caspase-like activity), catalytic dyad (H147/C202)	H_2_O_2_, heat shock, miltefosine, curcumin	Autophagy	González et al. (2007) [35]; Ambit et al. (2008) [2]; Zalila et al. (2011) [73]; Basmaciyan et al. (2018) [7]
*L. donovani*	*LdBPK_35180.1*	In promastigotes and axenic amastigotes	No processing	Acidocalcisomes (inhibited?)	Trypsine-like activity (no caspase-like activity), catalytic dyad (H147/C202)	H_2_O_2_	ND	Lee et al. (2007) [44]
*L. mexicana*	*LmxM.34.1580*	ND	ND	Mainly cytoplasmic	Catalytic dyad (H147/C202)	Miltefosine (not H_2_O_2_ and not camptothecin)	Negative regulator of intracellular amastigote proliferation	Castanys-Muñoz et al. (2012) [17]

### Aquaporin Li-BH3AQP

Li-BH3AQP is an aquaporin identified in *L. infantum*, encoded by the *LINF_220020300* gene, and that contains a Bcl-2 homology domain called BH3, found in Bcl-2 family proteins i.e. pro- and anti-apoptotic molecules [32]. This protein can bind to the anti-apoptotic molecule Bcl-X_L_ in mammalian cells and its expression in mammalian cells reduces cell viability [32]. In *L. infantum*, Li-BH3AQP, which is mainly perinuclear, has a pro-death activity dependent on key residues of the BH3 domain, reducing cell viability and inducing DNA fragmentation after the addition of pro-apoptotic stimuli such as staurosporine and antimycin A [32]. This protein also has pro-survival activity in nutrient deprivation or hypotonic stress conditions, this activity being independent of key residues of the BH3 domain [32]. Li-BH3AQP constitutes the first, and for the moment the only, non-enzymatic molecule identified as being involved in *Leishmania* cell death. However, the absence, at this time, of anti-apoptotic molecules identified, such as Bcl-X_L_, raises the question of the role of Li-BH3AQP in the parasite itself or in the host cell after release from the parasite [33]. Furthermore, additional experiments are required to evaluate the importance of this protein in cell death in other *Leishmania* species.

### Calpains

Calpains (calcium-activated papain-like proteases) are cysteine proteases that are calcium-dependent [34]. In mammals, typical calpains are involved in a broad range of cellular functions such as differentiation, proliferation, apoptosis, cell survival, cytoskeletal rearrangements and cell migration [58]. In *Leishmania*, like in other Trypanosomatids, several calpain-like proteins, much more than in mammals, have been described [27]. They include proteins with a well-conserved protease domain that are called CALP (calpain-like proteins) and short calpain-like proteins with a highly conserved N-terminal domain but that lack a protease domain, called SKCRP (small kinetoplastid calpain-related proteins) [27]. All *Leishmania* calpain-like sequences lack the C-terminal calmodulin-related calcium-binding domain found in most mammalian calpains [27]. Furthermore, the protease domain, when present, often lacks the catalytic triad cysteine/histidine/asparagine, which is critical for mammalian calpain catalytic activity, raising the question of their protease activity [27]. The presence of a high number of calpain-related proteins and their unique protein architecture suggest important *Leishmania*-specific functions. However, these functions are still a matter of debate, particularly concerning their involvement in parasite apoptosis. The involvement of calpain-like proteins in *Leishmania* cell death has been reported [3, 36]. However, calpain inhibitors do not have an effect on amphotericin B-induced apoptosis: they do not inhibit caspase-specific substrate PPL cleavage activity, do not decrease PI staining, and do not inhibit loss of mitochondrial membrane potential [43]. On the contrary, calpain inhibitor I inhibits apoptosis after NO treatment [36]. Additionally, in a contradictory manner, the same calpain inhibitor partly inhibits miltefosine-induced DNA fragmentation but does not prevent cell shrinkage [55]. Concerning calpain-related proteins, few experiments have been carried out to study their role in *Leishmania* apoptosis. One SKCRP, SKCRP14.1, which is downregulated in an antimonial-resistant strain, increases antimonial-induced apoptosis but protects from miltefosine-induced apoptosis [69]. Moreover, a yeast two-hybrid assay has shown that the calpain-like cysteine peptidase LmCALP27.2 encoded by the *LmjF.27.0500* gene interacts with the C-terminal domain of LmjMCA, which was confirmed by co-immunoprecipitation [16]. This protein, as well as six other *L. major* calpain-related proteins, was also over-represented in *L. major* cells treated with the pro-apoptotic drug miltefosine in comparison to untreated cells. More precisely, LmCALP4.1, LmCALP18.1, LmCALP20.1, LmCALP27.1, LmCALP27.2, LmCALP31.1 and LmSKCRP25.2 are from 3 to 49 times more expressed in the miltefosine-treated sample than in the untreated sample (Basmaciyan et al., submitted article). The fact that, among the calpain-related proteins identified as potentially involved in *Leishmania* apoptosis, only LmCALP4.1 contains a conserved catalytic triad suggests that these three aminoacid residues are not necessary for protein apoptotic activity. As a conclusion, at least some calpain-related proteins are involved in *Leishmania* apoptosis and the heterogeneity in the results could be due to the different apoptotic stimuli that have been used.

### Cathepsin B-like protein (CPC)

Cysteine proteinase C (CPC), a cathepsin B-like protein, has been shown to be a cell death effector in *Leishmania* [25]. This enzyme, encoded by the gene *LmjF.29.0820* in *L. major*, binds to the pan-caspase inhibitor Z-VAD-FMK [25]. Upon addition of an apoptotic stimulus such as miltefosine or heat shock, CPC is released from lysosomes to the cytosol, where it induces *Leishmania* apoptosis [25]. The rupture of the lysosomal membrane during *Leishmania* apoptosis could also explain the release of other cell death effectors, particularly other cathepsins like CPA and CPB [25, 74].

### LmjHYD36 and Lmj.22.0600

Recently, two new proteins have been identified as cell death effectors in *L. major*: LmjHYD36 and LmjF.22.0600 [6, 8]. Orthologs of both proteins are overexpressed during *L. infantum* differentiation from the promastigote (in the insect vector) to the amastigote (in the mammalian host) form [1, 51], during which autophagy occurs [10]. LmjHYD36 is encoded by the *LmjF.36.6540* gene. It contains the catalytic triad (cysteine, histidine and aspartic acid) characteristic of α/β-hydrolases and is highly conserved among *Leishmania* species [8]. In the TriTryp database, this protein is annotated as a potential endonuclease. However, the lack of a positive patch for DNA binding at the protein surface and the lack of clear nuclease activity *in vitro* challenge this function [8]. Overexpression of the *LmjHYD36* gene increases pentamidine-induced cell death, while its deletion inhibits cell death induced by pentamidine [8]. As a consequence, LmjHYD36 is an effector of the pentamidine-induced cell death pathway. LmjHYD36 is also involved in *Leishmania* cell death induced by curcumin and miltefosine but not by H_2_O_2_ [8].

LmjF.22.0600 is a potential acetyltransferase conserved among different *Leishmania* species. The corresponding gene is overexpressed in *L. major* cells after the addition of different pro-apoptotic drugs, and gene overexpression increases the apoptosis induced by curcumin and miltefosine [6]. On the contrary, deletion of *LmjF.22.0600* by CRISPR/Cas9 induces no significant difference concerning *Leishmania* cell death, suggesting that another protein can take over in the apoptotic pathway. This suggests the importance of LmjF.22.0600 for *Leishmania* cells [6]. Furthermore, LmjF.22.0600 is involved in the same apoptotic pathway as LmjMCA. In fact, *Lmjmca* expression is required for *LmjF.22.0600* expression and overexpression of *LmjF.22.0600* has no consequence on pentamidine-induced cell death, while pentamidine induces *L. major* apoptosis independently of LmjMCA [5, 6].

## Apoptosis pathways

**Figure 4:**
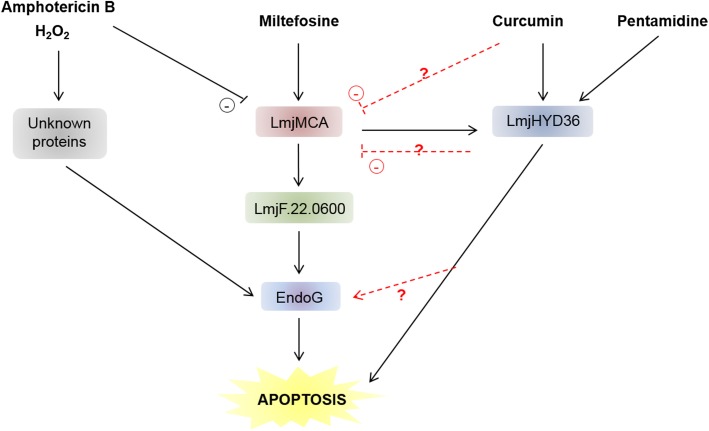
Proposed model of the different *L. major* cell death pathways. It is currently clear that different pro-apoptotic stimuli can induce different cell death pathways in *L. major*. While miltefosine induces LmjMCA and then LmjHYD36 and LmjF.22.0600 activation before activating EndoG, the amphotericin B and H_2_O_2_-induced cell death pathways involve unknown proteins. Curcumin and pentamidine, on the contrary, only activate LmjHYD36. The relationships between curcumin and LmjMCA and between LmjHYD36 and LmjMCA, on the one hand, and EndoG on the other, remain to be elucidated.

Like in mammals, where different apoptotic pathways have been described, for example caspase-dependent and caspase-independent pathways [13], several apoptotic pathways are being described in *Leishmania*. More precisely, concerning MCA, three apoptotic pathways have been identified in *L. major*: (i) a “classic” apoptotic pathway involving LmjMCA activation (induced by miltefosine), (ii) an original pathway involving LmjMCA inhibition (induced by amphotericin B, curcumin and H_2_O_2_) in which either LmjMCA inhibition directly induces apoptosis through its role in autophagy [16], autophagy inhibition inducing apoptosis [48], or its inhibition is not related to the apoptosis process, and (iii) an LmjMCA-independent pathway (induced by pentamidine). In the last case, several other proteases could be involved, such as calpains or cathepsins. Figure 4 illustrates these results.

Concerning LmjHYD36, as explained above, it is an effector of the pentamidine-induced cell death pathway but, on the contrary, it is not involved in the H_2_O_2_-induced apoptotic pathway [8]. Furthermore, LmjHYD36 is involved in cell death induced by curcumin in an LmjMCA-independent manner, curcumin inducing overexpression of the *LmjHYD36* gene in the wild-type strain as well as in the LmjMCA-deficient strain [8]. Lastly, while the *LmjHYD36* gene is overexpressed in the wild-type strain after the addition of miltefosine, no overexpression occurs in the LmjMCA-deficient strain, suggesting that LmjHYD36 acts downstream of LmjMCA in the miltefosine-induced apoptosis pathway [8]. As a consequence, H_2_O_2_ and amphotericin B could induce *L. major* apoptosis through proteins other than LmjMCA or LmjHYD36, not known at this time (Fig. 4). Miltefosine could induce the activation of LmjMCA that could activate LmjHYD36, both proteins being at the origin of the apoptotic phenotype (Fig. 4). On the contrary, curcumin would only activate LmjHYD36 (Fig. 4). Whether curcumin inhibits LmjMCA or has no direct action on LmjMCA remains to be determined. Finally, pentamidine may induce LmjHYD36 but not LmjMCA activation (Fig. 4).

As shown previously, LmjMCA is necessary for *LmjF.22.0600* expression and LmjF.22.0600 is not involved in the pentamidine-induced metacaspase-independent pathway [6]. The protein LmjF.22.0600 is thus downstream of LmjMCA in the proposed model (Fig. 4).

EndoG is activated by H_2_O_2_ [31] and the article from Chowdhury et al. [18] suggests that EndoG acts downstream of LmjMCA, allowing us to complete Figure 4. Finally, authors suggest that antimonials induce a cell death pathway different from the one induced by miltefosine in *Leishmania* [69]. However, other experiments must be conducted to identify the cell death pathways induced by antimonials and thus to complete our proposed model.

## Targeting *Leishmania* cell death

Because of the importance of cell death in the parasite biology, the specificity of the proteins and the cell death pathways involved, and the absence of several proteins in humans, proteins involved in *Leishmania* cell death appear to be a target of choice for the development of new therapeutic tools. To this end, proteins and signaling pathways involved must be identified in a more exhaustive manner. For this purpose, two methods can be proposed. The first one consists in identifying the proteins overexpressed during *Leishmania* cell death. This type of approach has been attempted by our group, highlighting the importance of calpains in *Leishmania* cell death (Basmaciyan et al., submitted article). However, these results must be supplemented and/or confirmed. For those who cannot do proteomics, proteins involved in *Leishmania* autophagy or at least in *Leishmania* differentiation from the promastigote to the amastigote form, during which autophagy occurs, appear a great source because of the close relationship between autophagy and apoptosis [48]. This approach allowed the recent identification of LmjHYD36 [8] and LmjF.22.0600 [6]. The second method consists in identifying protein partners or substrates. For instance, MCA being involved in *Leishmania* apoptosis, at least in some signaling pathways, the identification of MCA substrates will allow the identification of apoptosis effectors in *Leishmania*. Importantly, the cleavage sites of LmjMCA have been identified *in vitro*, different small peptides being good LmjMCA substrates: GGR, GRR, RR and VRPR [35, 49]. We thus listed the potential cleavage sites in all *L. major* proteins, as a source of potential LmjMCA substrates (Table S1).

Once proteins involved in *Leishmania* cell death have been identified, they can be targeted. Once again, several options are possible. First, owing to the necessity of *Leishmania* cell death in the biology of the parasite, the inhibition of this process thanks to specific inhibitors could constitute a new therapeutic approach. For instance, LmjMCA-binding peptides may be of interest for inhibiting *L. amazonensis* heat shock-induced death [57]. However, even though MCA seems to have a role similar to that of caspases in cell death, caspase inhibitors have no action on it [16, 44]. Specific MCA inhibitors must thus be used, which suggests weak cytotoxicity of the molecules towards host cells. Cross-talk between different cell death pathways, emphasized in other eukaryotes [29] and suggested in *Leishmania*, is nevertheless an issue for this approach, as inhibiting cell death implies inhibiting several pathways at the same time. As a consequence, activation of the apoptotic pathways seems more likely. Since the pathways are specific to the parasite, such activators would not affect mammalian host cells. Furthermore, *Leishmania* cell death by apoptosis would not induce a host immunological response, contrary to necrosis. To induce *Leishmania* cell death, proteins/peptides involved in parasite death can be fused to a cell permeant peptide after checking that it does not induce conformation changes. Catalytic proteins/peptides can also be encapsulated, for instance in lipidic vectors, allowing their passage through the different membranes to target the intracellular amastigotes and induce their death. This approach could even be applied for other intracellular infectious agents, such as *Plasmodium falciparum*.

## Conclusion

**Figure 5:**
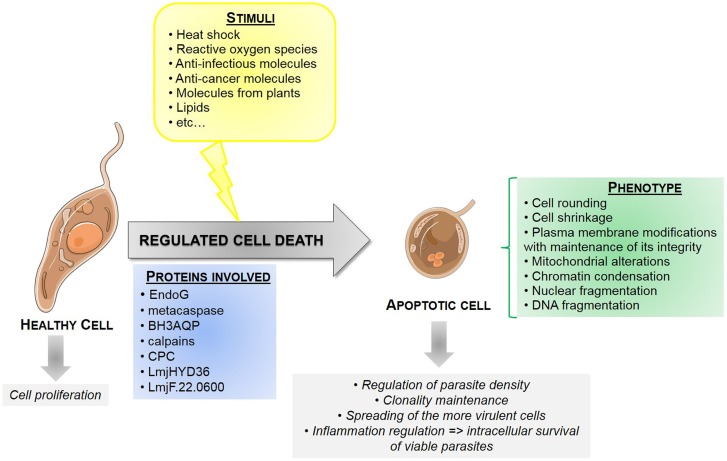
Schematic representation of *L. major* RCD. This schematization summarizes the physiological roles of *Leishmania* RCD, the phenotype of apoptosis, the pro-apoptotic stimuli and the proteins involved that are currently known.

The recent discovery of several proteins as cell death effectors indicates that *Leishmania* cell death is regulated and not incidental, as previously suggested [59]. However, the knowledge on *Leishmania* cell death is less advanced than on other eukaryotes, whether higher eukaryotes or ancestral eukaryotes, such as yeast. The morphological description of *Leishmania* cell death should notably be withdrawn to the benefit of functional descriptions, since morphology gathers very different features, from totally necrotic to totally apoptotic. This approach will be made possible by a better comprehension of the proteins and pathways involved. It is therefore important not to generalize but to study the consequences of one stimulus at a time in a given species, different stimuli inducing different cell death pathways. Figure 5 summarizes the data obtained on *Leishmania* apoptosis, from the potential inducers to the *in vivo* consequences, through the phenotypic features and the proteins involved. Other experiments must be carried out to confirm that this general scheme applies to all *Leishmania* species and, especially, *in vivo*, in physiological conditions.

In conclusion, proteins involved in *Leishmania* cell death are targets of choice for the development of new therapeutic tools, owing to their high parasite specificity and importance, while leishmaniases still constitute a global health issue with no satisfactory treatment.

## Supplementary materials

Table S1. Number of potential LmjMCA cleavage sites. Supplementary material is available at https://www.parasite-journal.org/10.1051/parasite/2019071/olm.
